# Transcriptome-wide identification of NAC (no apical meristem/Arabidopsis transcription activation factor/cup-shaped cotyledon) transcription factors potentially involved in salt stress response in garlic

**DOI:** 10.7717/peerj.14602

**Published:** 2022-12-19

**Authors:** Guang-Long Wang, Ya-Hong An, Cheng-Ling Zhou, Zhen-Zhu Hu, Xu-Qin Ren, Ai-Sheng Xiong

**Affiliations:** 1 School of Life Science and Food Engineering, Huaiyin Institute of Technology, Huaian, China; 2 State Key Laboratory of Crop Genetics and Germplasm Enhancement, College of Horticulture, Nanjing Agricultural University, Nanjing, China

**Keywords:** NAC transcription factors, *Allium sativum* L., Evolutionary analysis, Gene expression, Salt stress

## Abstract

Soil salinity has been an increasing problem worldwide endangering crop production and human food security. It is an ideal strategy to excavate stress resistant genes and develop salt tolerant crops. NAC (no apical meristem/Arabidopsis transcription activation factor/cup-shaped cotyledon) transcription factors have been demonstrated to be involved in salt stress response. However, relevant studies have not been observed in garlic, an important vegetable consumed in the world. In this study, a total of 46 *AsNAC* genes encoding NAC proteins were identified in garlic plant by transcriptome data. Phylogenetic analysis showed that the examined AsNAC proteins were clustered into 14 subgroups. Motif discovery revealed that the conserved domain region was mainly composed of five conserved subdomains. Most of the genes selected could be induced by salt stress in different tissues, indicating a potential role in salt stress response. Further studies may focus on the molecular mechanisms of the *AsNAC* genes in salt stress response. The results of the current work provided valuable resources for researchers aimed at developing salt tolerant crops.

## Introduction

Transcription regulation is a common manner to regulate gene expression in biological processes during plant development, as well as responses to adverse conditions (Yeger-Lotem et al., 2004). In this case, transcription factors (TFs) operate as important switches that bind to specific *cis*-regulatory elements, resulting in activation or repression of the target genes (Jin et al., 2014; Singh, Foley & Oñate-Sánchez, 2002). Therefore, identification and characterization of TFs is of vital importance to understand the transcriptional regulatory networks underlying different life activities. To date, a variety of plant TF families have been identified and classified based on their DNA-binding motifs. Some remarkable examples consist of MYB, AP2, WRKY, MADS, bHLH, and NAC (no apical meristem/Arabidopsis transcription activation factor/cup-shaped cotyledon) transcription factor families (Eulgem et al., 2000; Feller et al., 2011; Riechmann & Ratcliffe, 2000). The NAC family genes account for one of the largest plant-specific TF families (Nakashima et al., 2012). Owing to the rapid development of sequencing methods, acquirement of plant sequences is becoming more and more convenient. Genome-wide analysis has discovered 149 NAC TF members in the genome of rice (*Oryza sativa*) (Xiong et al., 2005), which are 142 in kiwifruit (*Actinidia eriantha*) (Jia et al., 2021), 81 in mung bean (*Vigna radiata*) (Tariq et al., 2022), 82 in lotus (*Nelumbo nucifera*) (Song et al., 2022), 87 in *Sesamum indicum* (Zhang et al., 2018), 112 in strawberry (Moyano et al., 2018), and 84 in *Salvia miltiorrhiza* (Zhang et al., 2021).

Generally, the structure of a typical NAC TF possesses a conserved DNA-binding domain at the N-terminus and a highly variable C-terminal region (Puranik et al., 2012). The conserved N-terminal domain is comprised of five subdomains (A–E) with about 150 amino acid residues, connected with DNA binding and protein–protein interactions (Sun et al., 2018). The C and D subdomains with net positive charges can allow the TF to bind to specific *cis*-acting DNA sequences (Chen et al., 2011; Ernst et al., 2004), whereas the A subdomain is in charge of TF dimerization, and the B and E subdomains may be responsible for the diverse functions of the NAC genes (Ooka et al., 2003; Puranik et al., 2012). The C-terminal transcriptional regulatory domain may act as either an activator or a repressor, and in some cases can exhibit protein-binding activity (Puranik et al., 2012). In addition, a transmembrane motif is observed in the C-terminal region of some NACs, which is associated with plasma membrane or endoplasmic reticulum membrane anchoring. By now, these specific NAC TFs have no more than 20 members in any single plant species, and were demonstrated to be induced by environmental signals (Lee et al., 2012; Seo et al., 2010).

Since the first NAC gene, *no apical meristem* (*NAM*), was identified from petunia in 1996, the NAC TFs have been proved to operate in developmental processes, as well as responses to adverse conditions (Guo et al., 2021; Nakano et al., 2015; Nuruzzaman, Sharoni & Kikuchi, 2013). Mutation in the *NAM* gene, the firstly identified NAC gene, resulted in stunted shoot apical meristem (SAM) in *Petunia* embryos (Souer et al., 1996). Similar functions were observed in mutants missing *CUC1* and *CUC2* genes in *Arabidopsis* (Aida et al., 1997). Further studies revealed that NAC transcription factors, VASCULAR-RELATED NAC-DOMAIN (VND) and NAC SECONDARY WALL THICKENING PROMOTING FACTOR1 (NST1) operate as master switches in secondary cell wall (SCW) formation in plants (Mitsuda & Ohme-Takagi, 2008; Nakano et al., 2015; Zhong, Richardson & Ye, 2007). Many NAC proteins are positive transcription regulators in response to abiotic stresses, while some are negative regulators (Sakuraba et al., 2015; Tran et al., 2004). In *Arabidopsis*, ANAC019, ANAC055 and ANAC072 act as transcription activators of stress-inducible genes, and overexpression of these genes led to enhanced drought tolerance (Tran et al., 2004). Genome-wide identification and expression analysis explored a group of NAC genes potentially functioning in drought stress in maize (Shiriga et al., 2014). Transcript levels of *GhNAC22* and *GhNAC34* in cotton (*Gossypium hirsutum*) was strongly induced by salt and drought stresses (Shah et al., 2014).

Garlic, domesticated and consumed all over the world, is a popular flavouring and green vegetable with medicinal properties (Atif et al., 2021; Kamenetsky et al., 2015; Xing et al., 2022). Garlic is rich in organosulfur compounds that contribute to garlic’s pungent smell and medicinal properties (Jones et al., 2004). In the past decades, China is one of the largest countries cultivating and producing garlic bulbs. However, due to the lack of genetic sequences, genetic studies and modern breeding are extremely restricted.

In the present study, a total of 46 NAC transcription factors were identified in transcriptome data with garlic clove samples from four time points under salt stress. Subsequently, phylogenetic relationships and conserved motif analysis were carried out. According to the results from evolutionary relationships and structural analysis, 11 NAC genes were selected for quantitative real-time PCR analysis. Transcription of these genes was determined in garlic tissues (clove, leaf, and root) and under salt stress. This study would shed light on functional characterization of NAC genes in garlic and provide a valuable resource for the improvement of plant stress tolerance.

## Materials and Methods

### Identification of the NAC family genes in garlic

To identify NAC genes potentially involved in salt stress in garlic, all of the genes from transcriptome results were searched against the PlantTFDB database (http://planttfdb.cbi.pku.edu.cn/) (Jin et al., 2017). The resulting protein sequences were further validated by InterProScan (http://www.ebi.ac.uk/interpro/) to check for the presence of NAC domains. Fragments without complete open reading frames and the proteins with very short amino acid sequences (<150 aa) were excluded. In addition, conserved motifs of NAC genes were explored using MEME (http://meme-suite.org/index.html) and visualized with TBtools software (Chen et al., 2020). The NAC genes and their encoding proteins were listed in Table S1. ExPASy program (http://web.expasy.org/protparam/) was introduced to analyze the predicted amino acid residues, molecular weight, and isoelectric points of NAC proteins. The transmembrane helix domains within NAC proteins were detected using the TMHMM server v.2.0 (http://www.cbs.dtu.dk/services/TMHMM/).

### Phylogenetic analysis of NAC proteins

ClustalX software was utilized to establish the multiple sequence alignments of NAC amino acid sequences with default settings. Phylogenetic analysis was performed utilizing the complete amino acid sequences of predicted NAC family transcription factors. MEGA5 software was applied to generate phylogenetic trees by using the Neighbour-Joining method (Kumar, Stecher & Tamura, 2016).

### RNA-seq based expression analysis of AsNAC genes in garlic

For identification and expression analysis of *AsNAC* genes, the RNA-seq data for garlic cloves collected at four time points under salt stress were used (Wang et al., 2019a). Genes with variation in FPKM (Fragments Per Kilobase Of Exon Per Million Fragments Mapped) values above two folds were recognized as differentially expressed genes, and *NAC* genes were identified by PlantTFDB database. The sequences were assessed and that lacked the whole NAC domain were removed. A heat map representing differential expression levels of *AsNAC* genes was constructed with HemI 2.0 software (https://hemi.biocuckoo.org/) (Ning et al., 2022).

### Plant material and stress treatments

The garlic cultivar ‘Cangshan siliuban’, widely cultivated in China, possesses 4 to 6 cloves, homogeneous and crisp texture, strong spicy taste, and high quality. It was salinity-treated and used for expression analysis in this study. Garlic plants were first grown in a container filled with a mixture of organic soil and vermiculite (1:1; v/v). After 10 d, the garlic seedlings were transferred to a hydroponic media with 1/2 Hogland nutrient solution. After rejuvenation and acclimation for a week, the seedlings were treated with 200 mM NaCl for 0, 1, 4, and 12 h. The garlic cloves, roots, and leaves from each time point were harvested, immersed in liquid nitrogen, and kept at −80 °C for RNA extraction.

### RNA isolation and quantitative RT-PCR analysis

Total RNA isolation was conducted on garlic clove, root, and leaf tissues using an RNAprep pure plant kit (Tiangen, Beijing, China) in accordance with the manufacturer’s instructions. Total RNA was DNase-treated and then used for reverse transcription using HiScript II Q RT SuperMix for qPCR (Vazyme Biotech, Nanjing, China). cDNA samples were diluted tenfold and stored at −20 °C until qRT-PCR analysis. The primers used for qRT-PCR were designed using the Primer 6.0 software (Table 1). All reactions were performed using ChamQ SYBR qPCR Master Mix (Vazyme Biotech, Nanjing, China) in a CFX96 Real-Time PCR Detection System (Bio-Rad, Hercules, CA, USA). The reaction conditions were set as follows: initial step of 30 s at 95 °C, followed by 40 cycles of 5 s at 95 °C, and 30 s at 60 °C. Melting curve analysis was followed to ensure the PCR product specificity. The raw values were shown in Table S2 and were directly utilized for gene expression normalization. The 2^−ΔΔCt^ method was used to generate the relative expression levels of each gene across all samples with *ACTIN* as the internal control (Livak & Schmittgen, 2001; Wang et al., 2019b).

**Table 1:** Primer sequences used for qRT-PCR.

Name	Gene ID	Forward primer (5′-3′)	Reverse primer (5′-3′)
*AsNAC1*	Unigene37735	GCAGAGAGCCGAAGAAGGAGAAA	CCGTCATACTGATCGCCGAAGTT
*AsNAC7*	CL4976	AACCACTACGGCAATGAAGAAGAGAC	ACGAACACGGCTGAGGCAAAC
*AsNAC9*	Unigene15235	GGGCAGAAGACCGATTGGGTTATG	TGAATCTGAATGAGCAGGCGAAGA
*AsNAC11*	Unigene42862	CTACACCATTGAACCAAGCATCTCC	GAGCACTTCATCATTAGCCACATTACA
*AsNAC17*	Unigene6942	CTCATACACCACCTAAGGAGGACTG	CCGAAGCATCCACCTAACATTGATTG
*AsNAC18*	Unigene32815	GCTACAGAAGCAGGATACTGGAAGG	GGTTTAGGAAAGTTAGCCTCAGCAGAT
*AsNAC19*	Unigene36960	CCGATCACCTCAGTTCAGCAGAA	GTCAACTTCCGTACCACTAGGAGTATT
*AsNAC20*	Unigene39811	GCAGATCACCTCAATTCATCCAATCC	CAACTAGATATGCTGTCCTGAGAACCA
*AsNAC25*	Unigene33777	CAAGAGAAGAAGAGATGGAGCAAGTCA	TGCCGAGGACAGAAGAAGAACCA
*AsNAC27*	CL10474	GCTTGGTACACTGCAACGGTAGTAA	TTGACTTCTCGGACTGGAGGATGG
*AsNAC29*	CL17133	AGAGACGCAGAAGCAGAATTGAATCT	GCAGAGGTAATGGACGACGAGTTC
*AsACTIN*	CL2365	TGCTCTGGATTATGAACAGGAACTTGA	CAATCATTGAAGGCTGGAACAACACT

### Statistical analysis

The data were expressed as the mean ± standard deviation (SD) and analyzed using SPSS 16.0 software at the 0.05 significance level by Duncan’s method.

## Results

### Identification of AsNAC genes from garlic

Based on the transcriptome results, a total of 46 non-redundant NAC genes were identified. All the selected AsNAC proteins possessed a conserved NAM domain at the N-terminus. The predicted AsNAC proteins varied from 192 (AsNAC22) to 934 (AsNAC27) amino acids in length, with relative molecular weights ranging from 22.10 kDa (AsNAC22) to 106.25 kDa (AsNAC27) (Table 2). The largest theoretical pI value was 9.44 (AsNAC9), whereas AsNAC41 showed the least pI value (4.57). Besides, seven AsNAC proteins (AsNAC7, AsNAC11, AsNAC30, AsNAC31, AsNAC32, AsNAC38, and AsNAC40) were assumed to be membrane-bound transcription factors (MTFs), since they contained transmembrane motifs at the C-terminal.

**Table 2:** List of all AsNAC genes information identified in the garlic transcriptome.

Name	Gene ID	Length (aa)	Molecular weight (kDa)	pI	Transmembrane regions
*AsNAC1*	Unigene37735	297	34.00	6.36	‒
*AsNAC2*	Unigene6332	789	89.31	6.22	‒
*AsNAC3*	Unigene37976	212	24.25	8.86	‒
*AsNAC4*	Unigene33030	297	33.81	8.08	‒
*AsNAC5*	Unigene38522	247	28.22	6.33	‒
*AsNAC6*	CL198	917	103.86	5.13	‒
*AsNAC7*	CL4976	425	49.11	5.17	392–414
*AsNAC8*	CL11812	242	27.50	9.42	‒
*AsNAC9*	Unigene15235	214	24.19	9.44	‒
*AsNAC10*	Unigene19445	276	31.66	8.51	‒
*AsNAC11*	Unigene42862	476	53.65	6.23	456–475
*AsNAC12*	Uingene36091	246	28.48	5.21	‒
*AsNAC13*	Unigene34286	239	27.79	5.81	‒
*AsNAC14*	Unigene77877	295	33.20	8.67	‒
*AsNAC15*	Unigene42432	278	31.63	8.31	‒
*AsNAC16*	Unigene27135	277	31.59	8.45	‒
*AsNAC17*	Unigene6942	258	29.48	5.24	‒
*AsNAC18*	Unigene32815	300	34.38	6.34	‒
*AsNAC19*	Unigene36960	364	41.17	4.89	‒
*AsNAC20*	Unigene39811	383	43.11	7.21	‒
*AsNAC21*	CL6159	295	33.53	8.60	‒
*AsNAC22*	Unigene33003	192	22.10	5.21	‒
*AsNAC23*	CL15358	298	34.58	5.92	‒
*AsNAC24*	Unigene33621	291	33.23	7.02	‒
*AsNAC25*	Unigene33777	222	25.26	5.20	‒
*AsNAC26*	CL2738	439	49.00	4.91	‒
*AsNAC27*	CL10474	934	106.25	5.90	‒
*AsNAC28*	CL14328	275	31.52	6.25	‒
*AsNAC29*	CL17133	320	37.01	5.63	‒
*AsNAC30*	Unigene15491	659	73.12	4.73	630–652
*AsNAC31*	CL14578	569	64.97	4.72	533–555
*AsNAC32*	CL16988	646	71.91	4.62	622–644
*AsNAC33*	CL8897	210	23.99	6.11	‒
*AsNAC34*	CL6035	258	29.65	6.00	‒
*AsNAC35*	Unigene36323	244	28.26	6.39	‒
*AsNAC36*	Unigene22191	415	47.13	7.08	‒
*AsNAC37*	Unigene5796	221	25.40	7.02	‒
*AsNAC38*	Unigene13443	586	66.68	4.65	552–574
*AsNAC39*	Unigene34492	286	32.28	5.73	‒
*AsNAC40*	CL17514	565	62.68	4.73	541–563
*AsNAC41*	Unigene43063	421	46.88	4.57	‒
*AsNAC42*	CL13253	278	31.63	8.31	‒
*AsNAC43*	Unigene83480	265	28.97	9.28	‒
*AsNAC44*	CL5657	258	30.27	6.00	‒
*AsNAC45*	Unigene10665	317	36.00	6.77	‒
*AsNAC46*	CL17458	238	27.68	6.00	‒

### Phylogenetic relationships and classification of NAC family TFs in garlic

**Figure 1:**
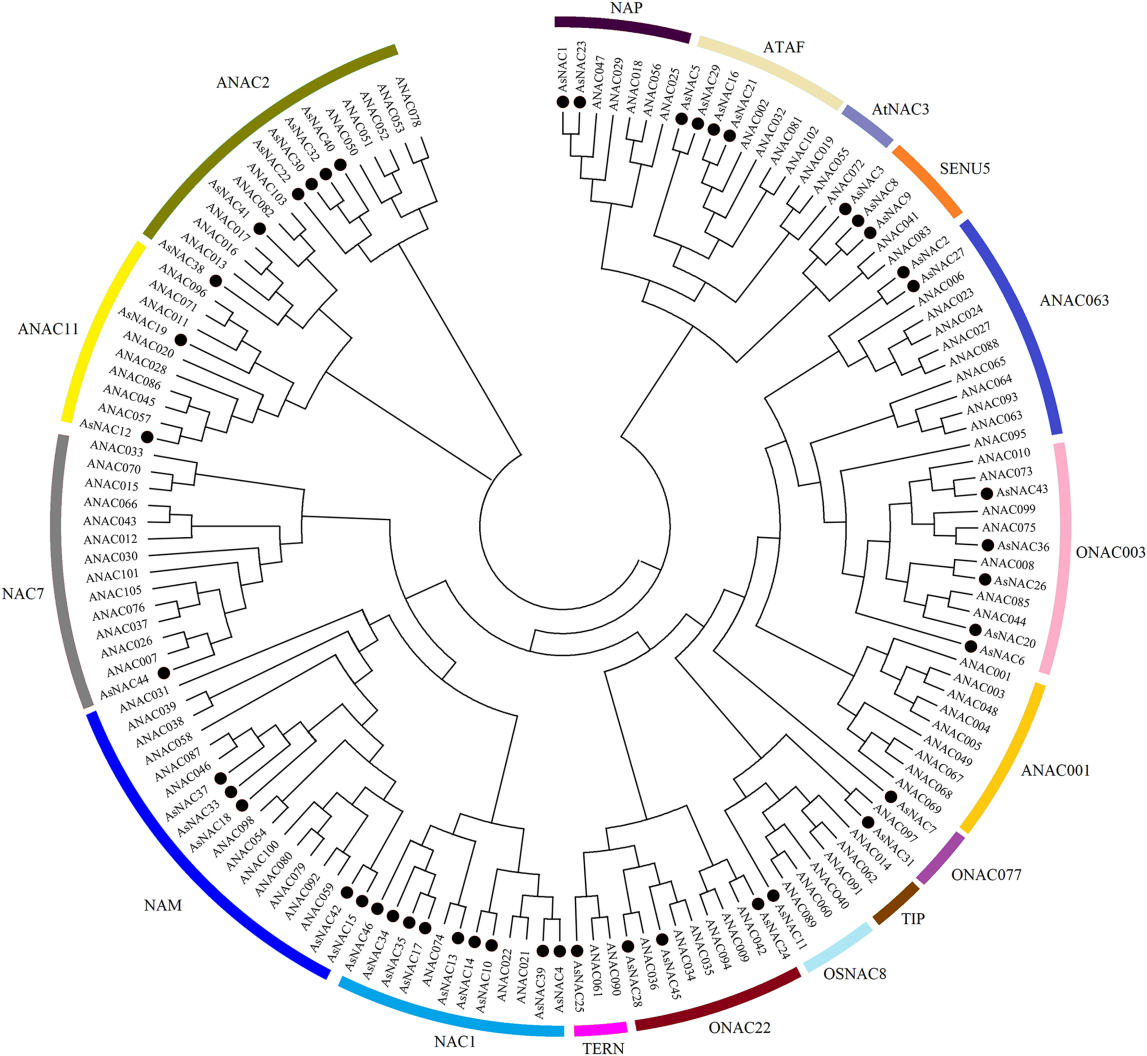
Phylogenetic tree of NAC proteins from *Arabidopsis thaliana* and *Allium sativum*. The alignment of NAC proteins was carried out by Clustal X 1.83, and the phylogenetic tree was established using MEGA 5.0 by the neighbor-joining (NJ) method with 1,000 bootstrap replicates. The black circles represent NAC proteins from garlic, whereas others indicate the NAC proteins from Arabidopsis.

NAC family transcription factors have been extensively characterized in Arabidopsis, a model organism of choice for research in plant biology. To elucidate the phylogenetic relationships of NAC family proteins in garlic and Arabidopsis, a phylogenetic tree was generated based on aligned NAC domains. The results indicated that 46 and 102 NAC transcription factors from garlic and Arabidopsis, respectively, were clustered into 17 subgroups. As shown in Fig. 1, AsNAC proteins were non-uniformly scattered in 14 subgroups. The NAC1 subgroup possessed the largest number of AsNAC proteins. By contrast, no AsNAC proteins were observed in TIP, ANAC001, and AtNAC3 subgroups.

### Gene structure and conserved motif analysis

**Figure 2:**
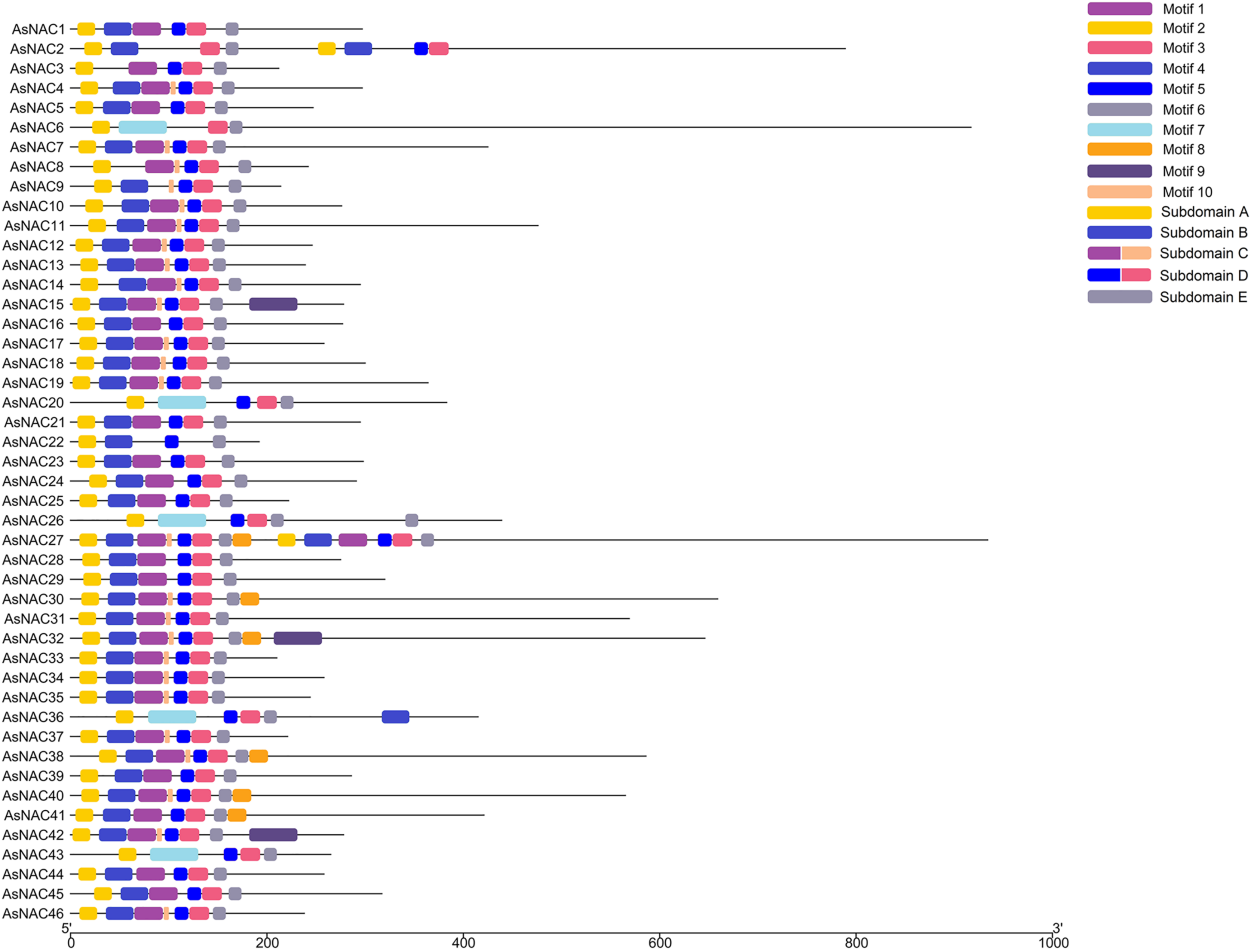
Discovery of conserved motif compositions of AsNAC proteins. Each motif is expressed by a color rectangle numbered, whereas black lines indicate non-conserved sequences.

**Figure 3:**
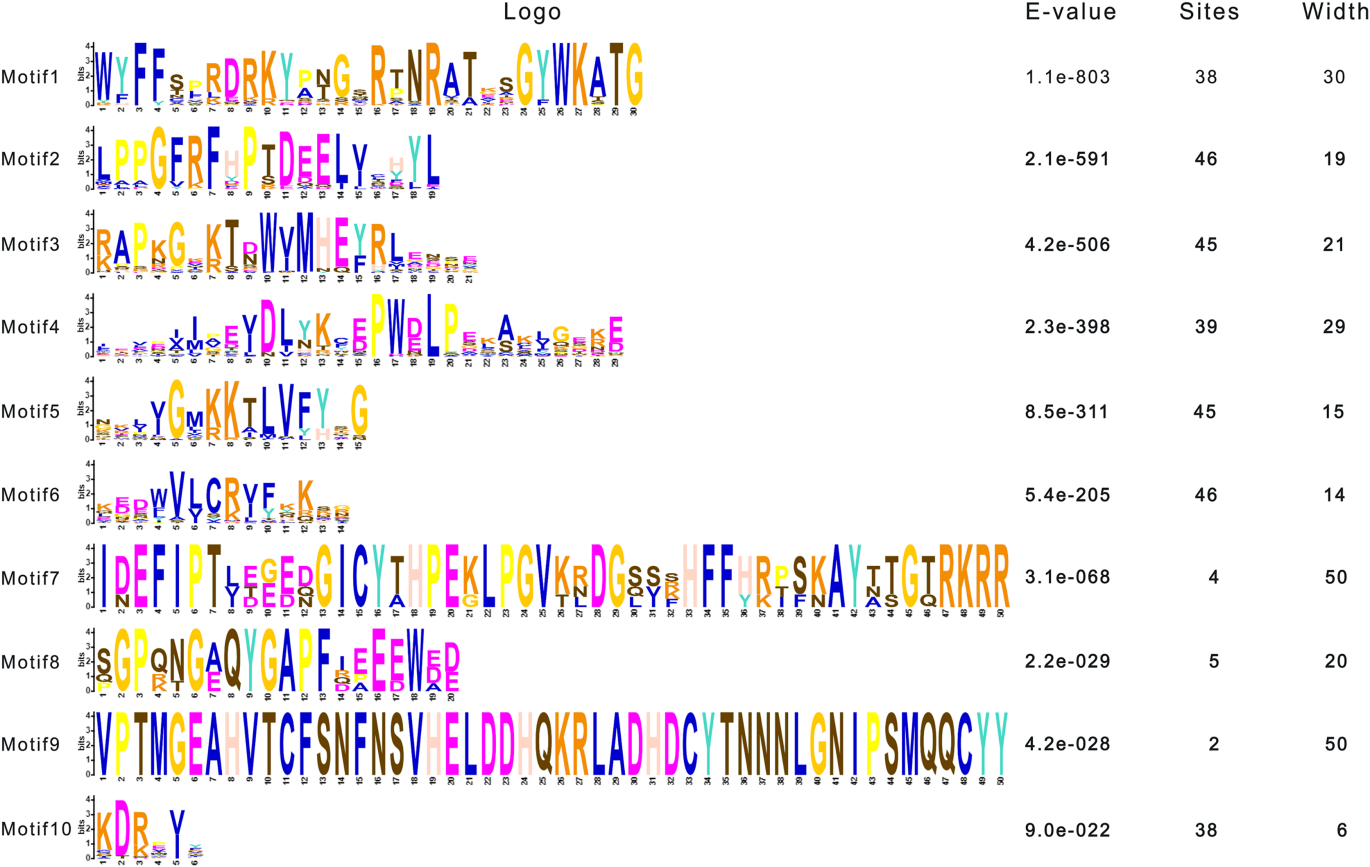
Sequence logos of NAC TF domains in *Allium sativum*. Residue height within a stack indicates the relative frequency at that position.

To dissect the sequence characteristics of AsNAC proteins, the conserved motifs of studied proteins were determined using the MEME software. A total of 10 conserved motifs were generated (Figs. 2 and 3). Motifs 2, 4, and 6 corresponded to the conserved subdomains A, B, and E, respectively, whereas subdomains C and D were composed of two motifs. Members with the same or similar motif structures tended to be close in evolutionary relationship (Figs. 1 and 2). Most of the examined AsNAC proteins displayed the complete A–E subdomains, which were primarily present at the N-terminal conserved domain region. The subdomains A and E existed in all protein sequences, indicating a highly conserved constituent of the NAC proteins.

### Expression patterns of AsNAC genes in garlic transcriptomes under salt stress

**Figure 4:**
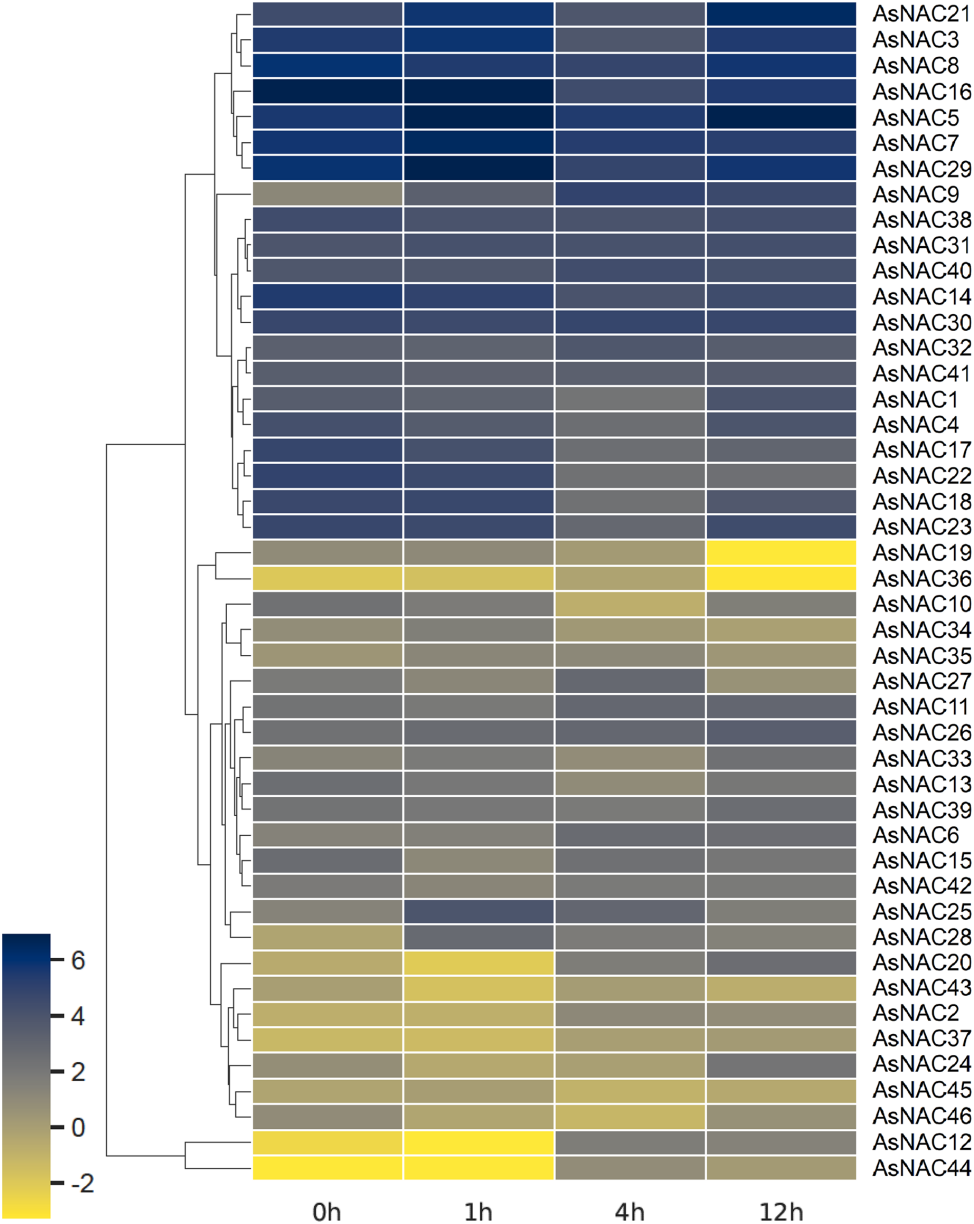
Heat map representing *AsNAC* genes at different time points of salt stress. The FPKM values of the RNA-seq data were log2 transformed, and heat map was generated using HemI 2.0 software (https://hemi.biocuckoo.org/).

RNA-seq based digital gene expression was derived to determine the expression patterns in garlic cloves under salinity stress. A heat map displaying differential expression at different time points was established on the basis of log2 transformed fragments per kilobase of exon per million mapped fragments (FPKM) values (Fig. 4). Most of the *AsNAC* genes examined were differentially expressed under salinity condition. Transcript levels of *AsNAC9*, *AsNAC12*, *AsNAC19*, *AsNAC20*, and *AsNAC44* underwent great alterations (> 10 fold) during salinity treatment. By contrast, some genes including *AsNAC30*, *AsNAC31*, and *AsNAC41* showed relatively stable expression under salinity condition (Fig. 4).

### Expression analysis of AsNAC genes in garlic tissues under salt stress

**Figure 5:**
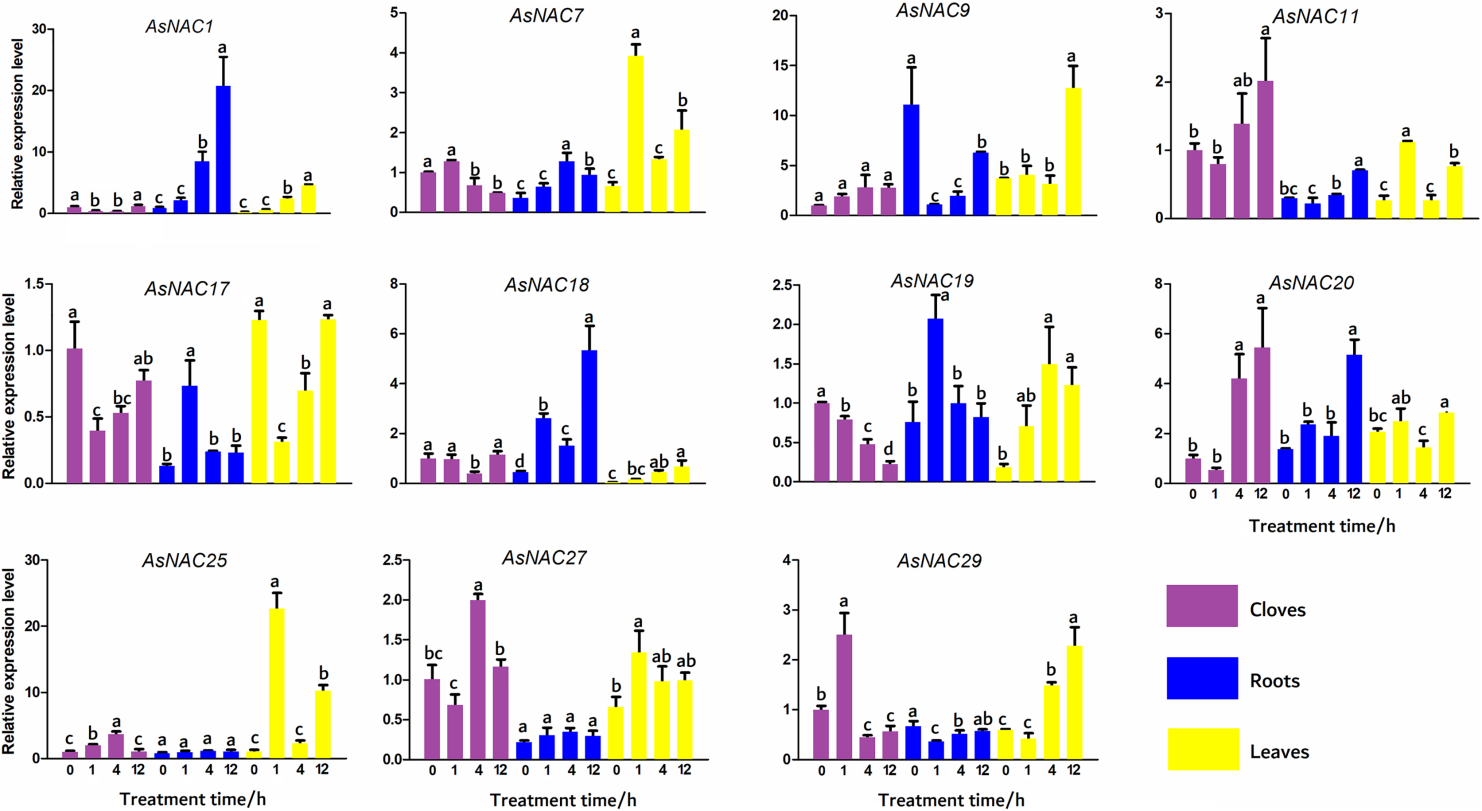
Expression patterns of *AsNAC* genes in garlic plants under salinity stress. Each bar is expressed by the means of three replicates ± standard deviation (SD). Different lowercase letters indicate significant differences at *P* < 0.05.

To validate the expression levels of the differentially expressed genes obtained from transcriptome data, *11 AsNAC* genes were randomly selected to detect their transcript levels in different tissues during salinity stress (Fig. 5). In the cloves, the expression patterns of most genes under salinity condition were well correlated with that in the transcriptome results. The expression levels of *AsNAC1* and *AsNAC17* showed a downward trend when exposed to salinity stress, and increased to the level before treatment at the last stage. *AsNAC7* were highly expressed at the first two stages and remained relatively low transcription at the latter two stages, whereas *AsNAC11* and *AsNAC20* showed a completely opposite pattern. Transcript levels of *AsNAC19* had been declining since salinity initiated. *AsNAC25* and *AsNAC27* displayed the largest mRNA abundance at 4 h after treatment, whereas *AsNAC29* were highly expressed at 1 h after salinity. In the roots, transcription of *AsNAC1* showed a continuously increasing trend with the extension of processing time. *AsNAC7* was stably expressed at the first two stages and increased its mRNA abundance at the third stage, followed by an evident decline. Transcript levels of *AsNAC9*, *AsNAC11*, and *AsNAC29* were dramatically decreased when salinity treatment was applied and ascended at 12 h after treatment. *AsNAC17* and *AsNAC19* exhibited the highest expression at 1 h and relatively lower expression levels at the other stages. *AsNAC18* and *AsNAC20* showed the highest and lowest mRNA levels at 12 h and 0 h, respectively, whereas *AsNAC25* and *AsNAC27* had no obvious changes in expression throughout the whole treatment. In the leaves, all of the examined genes were differentially expressed during salinity process. Salinity did not influence *AsNAC1* and *AsNAC29* transcription at the early two stages, followed by a continuous increase at the latter stages. *AsNAC7*, *AsNAC11*, and *AsNAC25* had similar expression patterns with the highest expression at 1 h and lowest at 0 and 4 h. *AsNAC9* was highly expressed at 12 h after salinity and demonstrated relatively lower transcript levels at the remaining stages. When salinity initiated, transcription of *AsNAC17* underwent a sharp decline, followed by a continuous increase at the latter stages (Fig. 5).

## Discussion

Salinity refers to the accumulation of high concentrations of salt in the soil, which impedes the growth and development of plants. It is a growing problem in the world that poses an increasing threat to crop yield and quality (Arif et al., 2020; Zhang et al., 2022). Abuse of chemical fertilizer and unreasonable irrigation results in excessive accumulation of salt in the soil and ultimately leads to the decline of arable property of soils (Amin et al., 2021). When exposed to salt stress, plants go through both ionic stress and osmotic force (Zhao et al., 2021). Osmotic stress is caused by the elevated sodium ions in the soil, bringing about the decline of water absorption capacity and making difference on a series of downstream physiological processes within plants. Some plants, such as halophyte species, can tolerate a high concentration of salt (Ventura et al., 2015). However, most plants, especially edible or exploitable crops, can only grow normally at a low level of salt. Therefore, many researches are devoted to reducing or counteracting the impact of salt damaged soil on crop growth and yield formation through physical, chemical, or molecular means. NAC family proteins are one of the largest classes of plant transcriptional regulators and play crucial roles in plant response and resistance to environmental stimuli (Ma et al., 2021; Puranik et al., 2012; Shao, Wang & Tang, 2015). However, the roles of NAC members in garlic plants under salt stress still remain elusive.

Previous studies have screened potential NAC transcription regulators involved in salt stress by transcriptome (Frosi et al., 2021; Wang et al., 2022). Here, a total of 46 NAC genes were identified from garlic transcriptome (Wang et al., 2019a). Most of them were differentially expressed when exposed to salt stress, indicating a crucial role of NAC transcription factors in plant response to salt stress in garlic. The evolutionary relationships, protein structures, and transcript levels of the studied genes in different tissues under salt stress were extensively investigated to comprehensively understand the NAC transcription factors in garlic.

NAC transcription factors commonly have a variable C-terminal and a highly conserved domain at the N-terminal, which is divided into five subdomains (A, B, C, D, and E) (Liu et al., 2018). In this study, most of the AsNAC proteins had all of the five subdomains, whereas some domain regions were incomplete in the minority proteins. Similar results were also observed in some previous studies (He et al., 2022; Yang et al., 2022), indicating that some NAC proteins possessed preternatural structures and unique functions. It is reported that subdomains A, C, and D are more conserved than B and E (Ooka et al., 2003; Wang et al., 2016). As mentioned above, C and D subdomains were responsible for the access to binding to the downstream genes, whereas A is involved in dimerization. Therefore, the difference in conservatism may be due to different functions they shall take on. In recent years, NAC MTFs have been demonstrated to be associated with response to abiotic stresses (Sun et al., 2022; Yan et al., 2021). Here, seven membrane-bound AsNAC proteins harboring α-helical transmembrane motifs at the C-terminal were identified using the TMHMM web server, indicating potential roles of these genes in abiotic stress response.

Numerous evidence has pointed out the roles of NAC proteins in abiotic stress response. Overexpression of pepper *CaNAC064* in Arabidopsis resulted in enhanced tolerance to cold stress as compared to wild-type plants (Hou et al., 2020). Similar results were also observed for *HuNAC20* and *HuNAC25*, two novel NAC genes from pitaya (Hu et al., 2022). *AfNAC1* from *Amorpha fruticosa* may have a part in photosystem regulation and indirectly confer plant resistance to drought stress (Li et al., 2022). By contrast, *TaSNAC4-3D* negatively regulated drought tolerance by bringing about increased oxidative damage and programmed cell death in wheat (Ma et al., 2022). Compared with control lines, transgenic tomato plants overexpressing *Prunus persica PpNAC56* could accumulate more osmoregulatory substances when exposed to high temperature and thus were more tolerant (Meng et al., 2022). The functions of NAC transcription factors in salt stress have also been extensively studied. *GmNAC06*, a NAC transcription factor from soybean, could alleviate or avoid the negative effects resulting from salinity by regulating proline and glycine betaine accumulation, ionic homeostasis, and plant type (Li et al., 2021). In Arabidopsis, a member of ATAF subfamily, ATAF1/ANAC002, increased the transcription of stress-associated genes, and thus improving plant tolerance to salt (Liu, Sun & Wu, 2016). By contrast, AtNAP/ANAC029, a positive character in senescence, negatively regulated salt stress response (Seok et al., 2017). AtNAC2/ANAC092 was demonstrated to be involved in salt-promoted senescence (Balazadeh et al., 2010). We speculated that the identified AsNAC proteins with high evolutionary relationship to that in Arabidopsis may have similar functions. Therefore, biochemical and molecular experiments are required to further identify the roles and mechanisms of AsNAC in response to salt and other abiotic stresses. Our findings provided a fundamental understanding of NAC proteins in garlic, and would facilitate breeding salt-tolerant crops.

## Conclusions

Based on the transcriptome data, 46 *AsNAC* genes were identified. Phylogenetic analysis, motif discovery, MTF characterization, and gene expression shed light on the functional analysis of AsNAC proteins. In summary, AsNAC proteins examined possessed high conservatism during evolution and could be induced by salinity stress, which could be potentially introduced to breed plants with enhanced resistance against stress conditions. However, it should be noted that the current work is just the beginning for functional studies of *AsNAC* genes on salt stress response, and further studies are required to focus on the biological functions of *AsNAC* genes and their specific molecular mechanisms.

## Supplemental Information

10.7717/peerj.14602/supp-1 Supplemental Information 1 Nucleotide sequences of the AsNAC genes and their predicted amino acid sequences.

10.7717/peerj.14602/supp-2 Supplemental Information 2 Raw data of examined genes by qRT-PCR in garlic samples under salt stress.

## Additional Information and Declarations

### Data Availability

The following information was supplied regarding data availability: The raw measurements are available in the Supplemental Files.
